# Parental involvement in the war in Croatia 1991-1995 and suicidality in Croatian male adolescents

**DOI:** 10.3325/cmj.2012.53.244

**Published:** 2012-06

**Authors:** Tomislav Franić, Goran Kardum, Iris Marin Prižmić, Nevia Pavletić, Darko Marčinko

**Affiliations:** 1 University of Split School of Medicine, University Hospital Split, Department of Psychiatry, Split, Croatia; 2 University of Split, Faculty of Philosophy, Split, Croatia; 3 National Institute of Mental Health, Bethesda, MD, USA; 4 University of Zagreb School of Medicine, University Hospital Zagreb, Department of Psychiatry, Zagreb, Croatia

## Abstract

**Aim:**

To investigate the association between parental war involvement and different indicators of psychosocial distress in a community sample of early adolescents ten years after the war in Croatia 1991-1995.

**Methods:**

A total of 695 adolescents were screened with a self-report questionnaire assessing parental war involvement, sociodemographic characteristics, and alcohol and drug consumption. Personality traits were assessed with the Junior Eysenck Personality Questionnaire; depressive symptoms with the Children’s Depression Inventory (CDI); and unintentional injuries, physical fighting, and bullying with the World Health Organization survey Health Behavior in School-aged Children. Suicidal ideation was assessed with three dichotomous items. Suicidal attempts were assessed with one dichotomous item.

**Results:**

Out of 348 boys and 347 girls who were included in the analysis, 57.7% had at least one veteran parent. Male children of war veterans had higher rates of unintentional injuries (odds ratio [OR], 1.2; 95% confidence interval [CI], 0.56 to 2.63) and more frequent affirmative responses across the full suicidal spectrum (thoughts about death – OR, 2.1; 95% CI, 1.02 to 4.3; thoughts about suicide – OR, 5; 95% CI, 1.72 to 14.66; suicide attempts – OR, 3.6; 95% CI, 1.03 to 12.67). In boys, thoughts about suicide and unintentional injuries were associated with parental war involvement even after logistic regression. However, girls were less likely to be affected by parental war involvement, and only exhibited signs of psychopathology on the CDI total score.

**Conclusion:**

Parental war involvement was associated with negative psychosocial sequels for male children. This relationship is possibly mediated by some kind of identification or secondary traumatization. Suicidality and unintentional injuries are nonspecific markers for a broad range of psychosocial distresses, which is why the suggested target group for preventive interventions should be veteran parents as vectors of this distress.

War represents a major stressor that can have long-lasting adverse influences on mental health (1). Milliken et al showed that upon the return from the Iraq war 42.7% of US combat veterans required mental health treatment (2). Most frequent psychopathological consequences of combat exposure are posttraumatic stress disorder (PTSD), anxiety, depression, and psychosomatic complaints (3,4). During the war in Croatia 1991-1995, more than 300,000 people were recruited to army service (5). Among Croatian war veterans, one of the most prevalent psychiatric diagnoses and the most common disorder comorbid with PTSD is depression (6-8). Veterans’ psychological distress inevitably impacts those with whom they interact (9). In fact, there is an association between psychopathological disturbances and a reduced effectiveness in parenting, perhaps because of veterans’ disrupted social functioning, emotional withdrawal, and decreased desire to interact with the children (9-11). Data on the influence of combat-related depression are scarce. Nonetheless, there is a significant body of evidence suggesting that “civilian” depression may negatively influence parenting behaviors (12,13) and that maternal mental health status following war affects children's adjustment (14). Further, psychological disturbances in war veterans could have a negative horizontal impact on their wives (15,16), and even a long-lasting vertical influence on children and further generations (17,18). Since psychiatric disorders associated with war exposure are categorical and not dimensional (19), it is possible that even more veterans exhibit non-specific, sub-threshold psychological problems, which could negatively impact their social, familial, and parenting roles. In addition to the direct individual consequences, soldiers’ absence during deployment might exert negative influence on the structural stability of the community, which is critical for the welfare of youth because it produces consistency and continuity in social relationships. Thus, structural stability helps build trust, enhances social support, and facilitates social control through commitment to community values and norms (20). War also creates a situation of social-norm disintegration, leading to social anomie and an increase in suicidal phenomena (21), which was of particular interest for this investigation. The aim of this study was to explore the association between parental war involvement and mental health problems in children, including depression, risky behaviors, sleep related problems, and suicidal ideation and attempts.

## Methods

### Participants

The study was administered in Splitsko-dalmatinska County during the 2005-06 school year. The targeted population comprised early adolescents, 11 years of age or older, which corresponds to the fifth grade of elementary school. The fifth grade could be a potentially stressful school period because of greater school pressure, more subjects, and new teachers. Therefore, the study sample consisted of only sixth grade students. According to data from the Split-dalmatinska County Direction for Education, there were 3172 sixth grade students in 42 elementary schools in the 2005-06 school year. The study began 3 months after the beginning of the school year and lasted till 3 weeks before the end to minimize the effect of the school pressure at the end of the school year. Initially, all 42 schools were invited to participate. However, because of the study time limitations, only the 14 schools that responded in a timely manner, with 840 students, were included, which represented about 26% of all sixth-grade students in the observed area. This could be a source of possible bias, but the size of the sample and the fact that the schools are public, obligatory, and that students are distributed according to the place of residence might compensate for this, and the sample can be considered as representative of the population.

### Measures

*Parental war involvement.* Parental war involvement was assessed by two dichotomous items: “Did your father participate in the Homeland War?” and “Did your mother participate in the Homeland War?” Participants were informed that parental war involvement included participation in the Croatian military or police forces. Data about the nature or intensity of combat engagement were not assessed. Nevertheless, all military and police personnel were exposed to war activities, even if they were not deployed, because considerable parts of the county were part of the combat zone and the city itself was attacked several times.

*Sociodemographic questionnaire.* The sociodemographic questionnaire was designed for the purposes of this study and assessed the following variables: age, sex, family education, background, and socioeconomic characteristics (parental education and employment, home ownership, perceived family standard), household structure (living in complete primary family, number of children in family, birth order, number of siblings), self-perceived family relationships, family cohesion, and parental control rated on a five-point Likert scale (relationship with mother, relationship with father, perceived relationship between parents, cohesion with family, and perceived parental control).

*Behavioral characteristics.* The behavioral characteristics questionnaire assessed school motivation and achievement (school motivation, last year school grade), religiosity (declarative religion and frequency of attendance to religious ceremonies), and risk behaviors. Four types of risk behaviors were assessed using World Health Organization survey Health Behavior in School-aged Children (22): unintentional medically attended injury in the past twelve months; physical fighting in past twelve months; and involvement in bullying behavior as victim, bully, or both. Unintentional injuries were measured with one question. Participants were asked how many times in the last twelve months they had incurred an injury (none, once, twice, three times, four times, or more). The following elaboration of the question was also included: “Many young people incur injuries while playing sports, or through street fights and domestic violence. Injuries can also include poisoning and burns. However, diseases (such as the flu or measles) are not considered injuries.”

*Personality characteristics.* Personality characteristics were assessed using the Croatian version of the Junior Eysenck Personality Questionnaire (JEPQ), a self-report personality scale (23,24). The JEPQ contains 81 dichotomous (ie, yes/no) items that can be allocated into three broad dimensions of personality: neuroticism (N), extraversion-introversion (Ex-I), and psychoticism (P). JEPQ also contains the Lie Scale (L), which measures socially desirable responses and serves as a control instrument (a lower score indicates greater interpretability of data). There were also attempts at constructing a criminality (C) scale consisting of 40 JEPQ items, which highly correlated with antisocial behaviors. According to Croatian standardization data, Cronbach coefficients were acceptable: Ex-I (α = 0.78); N (α = 0.79); P (α = 0.61); and L (α = 0.88) (23).

*Depressive symptoms.* Depressive symptoms were assessed using the Children’s Depression Inventory (CDI) for children aged 7-17 years, a self-rating scale that was developed on the basis of the Beck Depression Inventory (25,26). It consists of 27 items scored on a three-point scale (0 absent, 1 moderate, and 2 severe), reflecting a growing severity of symptoms, with the total score ranging from 0 to 54. Kovacs et al suggest a 19-point cut-off as the ideal threshold for discriminating children at risk of depression (25,26). The scores can also be interpreted by dividing responses into five categories: negative mood, interpersonal problems, ineffectiveness, anhedonia, and negative self-esteem (25,26). Several studies carried out in North America and Europe (including Croatia) investigated the psychometric characteristics of the scale, and corroborated its reliability and validity (25,27-30). According to Croatian standardization data, the Cronbach coefficient was satisfying (α = 0.82) (29).

*Suicidality.* Suicidal ideation was assessed with three dichotomous items: “I often think about death;” “I wish I was dead;” and “I often think about killing myself.” Suicide attempts were addressed with one dichotomous item “I have tried to kill myself.”

*Sleep-related problems.* This investigation was part of a broader assessment of suicidal ideation (31), and a standardized measure of sleep problems was not included in the original data set. Similar to other studies (32,33), we formed a composite measure of perceived sleep-related problems by combining items about sleep-related problems from the JEPQ (“Do you find it hard to sleep at night because you are worrying about things?”), CDI (“It is hard for me to fall asleep at night”), and two additional dichotomous questions (“I often was not able to fall asleep because of worrying” and “At times I was not able to stay asleep because of worrying”). Items were coded as 0 for “No” and 1 for “Yes,” except the CDI item that was scored 0 for “Never,” 1 for “Sometimes,” and 2 for “Often/always.” To standardize the total sleep-related problems score, the CDI responses were dichotomized so that a response of either “Sometimes true” or “Often/always true” was coded as “Yes.” The composite sleep-related problems measure was calculated by summing those responses. This score mainly assessed difficulties in initiating or maintaining sleep. A higher score indicated more difficulties. According to the total score, we divided the results into 3 categories: 0 scores, 1-2 scores (“moderate” sleep problems), and 3-4 scores (“severe” sleep problems). Internal consistency was computed for the total sleep-related problems scale and was acceptable (α = 0.72).

### Design and procedures

The study protocol was approved by the Ethics Committee of Splitsko-dalmatinska County Direction of Education. All permissions were obtained from school authorities, as well as informed consent from participants and parents. Questionnaires were administered in classrooms (up to 25 students) in the presence of at least one member of the study team and one member of the school staff, usually a psychologist. The estimated time for completion was 60 minutes. The aims of the study and nature of procedures were explained and manuals at the beginning of each section of the questionnaire provided appropriate instructions. Students were assured that the survey was anonymous and were asked to complete the questionnaires independently. After the entire class had completed the questionnaires, they were collected and analyzed.

### Statistical analysis

Statistica, 7.1 software package (StatSoft, Inc.,Tulsa, OK, USA, 2005) was used to perform statistical analysis of the data. Differences between categorical variables (sociodemographic, personal, and behavioral characteristics) were estimated by χ^2^ test, whereas differences between two samples on continuous variables (personality traits and depressive symptoms on CDI) were estimated by t-test for independent samples. The comparison was done for each sex separately. Interpretation of multivariate effects was performed by regression analysis. Parental war involvement was used as the dependent variable, and only the factors found to be significant in explorative bivariate analyses were included in the logistic regression. Statistical values were considered significant at *P*<0.05.

## Results

The final number of the study participants was 695 since 20 pupils (2.4%) were absent at the time of the survey, 17 students (2.0%) were excluded because they did not complete the suicidal items or J-EPQ, and additional 128 (15.2%) did not answer the question on parental war participation, so they were all excluded from the analysis. Among 695 pupils included in study, 401 (57.7%) had veteran parents (212 boys and 189 girls). In 22 (6.3%) boys and 22 (6.3%) girls, both parents were war veterans. The mean age of participants was 12.2±0.33 years. After preliminary analysis, there were no differences between adolescents with one or both veteran parents, so they were analyzed jointly.

Both groups were comparable according to overall sociodemographic characteristics, with significant differences in only a few examined variables (only those data that were significant for any sex were presented in Table 1).

**Table 1:** Association of socio-demographic, behavioral variables, and suicidality with parental war participation

Parental war participation	Boys				Girls			
	no (n, %)	yes (n, %)	odds ratio	95% confidence intervals	no (n, %)	yes (n, %)	odds ratio	95% confidence intervals
Father employment:								
yes	122 (42.4)	166 (57.6)	1		130 (47.8)	142 (52.2)	1	
no	9 (18.4)	40 (81.6)	3.27*	1.53-6.98	21 (32.8)	43 (67.2)	1.88^†^	1.06-3.33
Home ownership:								
yes	109 (36)	194 (64)	1		137 (45.2)	166 (54.8)	1	
no	25 (65.8)	13 (34.2)	0.29^†^	0.14-0.59	17 (44.8)	21 (55.2)	1.02	0.52-2.01
School motivation:^‡^								
1	100 (38.5)	160 (61.5)	1		140 (47.8)	153 (52.2)	1	
2	35 (40.7)	51 (59.3)	0.91	0.55-1.5	16 (31.4)	35 (68.6)	2^†^	1.06-3.78
Unintentional injuries:								
never	56 (33.1)	113 (66.9)	1		90 (46.4)	104 (53.6)	1	
1-2 times	69 (48.9)	72 (51.1)	0.52	0.33-0.82	54 (46.2)	63 (53.8)	1.01	0.64-1.6
3 times and more	11 (28.9)	27 (71.1)	1.22^†^	0.56-2.63	14 (38.9)	22 (61.1)	1.36	0.66-2.8
I often think about death:								
no	125 (41.1)	179 (58.9)	1		139 (46)	163 (54)	1	
yes	11 (25)	33 (75)	2.1^†^	1.02-4.3	19 (42.2)	26 (57.8)	1.17	0.62-2.2
I often think about killing myself:								
no	132 (41.8)	184 (58.2)	1		150 (46.7)	171 (53.3)	1	
yes	4 (12.5)	28 (87.5)	5.02*	1.72-14.66	8 (30.8)	18 (69.2)	1.97	0.83-4.67
I have tried to kill myself:								
no	133 (40.4)	196 (59.6)	1		153 (45.8)	181 (54.2)	1	
yes	3 (15.8)	16 (84.2)	3.62^†^	1.03-12.67	5 (38.5)	8 (61.5)	1.35	0.43-4.2

*P<0.005.

†*P* <0.05 (Pearson χ^2^ asymptotic significance 2-sided).

‡Originally there were 4 categories (“want to be the best” and “want to be among the good students” coded as 1; “neither good or bad” and “don’t care “coded as 2).

Sons of war veterans were likely to have an unemployed father (χ^2^_1_=10.146; *P*=0.001) and a parent with home ownership (χ^2^_1_=12.583; *P*=0.000). Daughters of war veterans were more likely to have an unemployed father than daughters of non-veterans (χ^2^_1_= 4.7; *P*=0.021). There were no other significant differences in the remaining sociodemographic characteristics. Only father’s employment significantly differed from 2001 census data for the general population (34).

Sons of war veterans reported more unintentional injuries (χ^2^_2_= 9.9;*P*=0.007) than sons of non-veterans. Daughters of war veterans were more likely to exhibit decreased school motivation than daughters of non-veterans (χ^2^_1_= 4.72; *P*=0.030).

Sons of war veterans were more likely to answer affirmatively on the items: “I often think about death” (χ^2^_1_= 4.19; *P*=0.041), “I often think about killing myself” (χ^2^_1_= 10.46; *P*=0.001), and “I have tried to kill myself.” (χ^2^_1_= 4.58; *P*=0.032) than sons of non-veterans. There were no differences on suicidal items between daughters of veterans and daughters of non-veterans (Table 1).

There was no significant difference in the scores on the Psychoticism, Extroversion-Introversion, Criminality, or Lie scale between children of war veterans and children of non-veterans. There was no also significant difference between boys and girls (Table 2).

**Table 2:** Junior Eysenck Personality Questionnaire and Children Depression Inventory scores in male and female adolescents regarding parental participation in war

	Boys				Girls			
Veteran parent	no (mean±standard deviation)	yes (mean± standard deviation)	*P*	95% confidence interval	no (mean± standard deviation)	yes (mean± standard deviation)	*P*	95% confidence interval
Neuroticism	8.81±4.22	8.83±4.29	0.964	(-0.9-0.94)	10.06± 4.55	10.60±4.56	0.272	(-0.43-1.51)
Psychoticism	3.57±2.76	3.95±2.96	0.232	(-0.24-1.0)	2.49±2.17	2.47±2.2	0.923	(-0.49-0.44)
Extroversion-Introversion	17.63±3.22	17.61±3.33	0.964	(-0.73-0.69)	17.13±3.21	17.61±3.37	0.182	(-0.22-1.18)
Desirable response	9.41±4.24	9.20±4.79	0.672	(-1.20-0.78)	10.03±4.83	10.14±4.65	0.826	(-0.89-1.12)
Criminality	18.97±5.32	19.28±6.06	0.628	(-0.94-1.56)	18.07±5.07	18.98±5.24	0.101	(-0.18-2.01)
Children’s Depression Inventory total	8.24±8.47	9.25±9.13	0.305	(-0.92-2.92)	6.82±7.11	8.66±9.97	0.052	(-0.02-3.71)
Negative mood	1.92±2.12	2± 2.28	0.726	(-0.4-0.57)	1.84± 2.10	2.15±2.53	0.209	(-0.18-0.82)
Interpersonal difficulties	1.24±1.6	1.49±1.9	0.195	(-0.13-0.64)	0.78±1.19	1.02±1.55	0.117	(-0.06-0.53)
Ineffectiveness	1.46±1.76	1.59±1.85	0.488	(-0.25-0.53)	1.04±1.39	1.35±1.73	0.069	(-0.02-0.65)
Anhedonia	2.52±2.95	2.84±2.92	0.317	(-0.31-0.96)	2.11±2.2	2.84±3.19	0.017*	(0.13-1.31)
Negative self-esteem	1.11±1.69	1.31±1.85	0.307	(-0.19-0.59)	1.04±1.79	1.3±2.24	0.244	(-0.18-0.69)

*P < 0.05.

Only daughters of war veterans had significantly higher scores on the CDI anhedonia subscale, whereas the total CDI score had borderline values (*P*=0.052) (Table 2).

There were no significant differences in sleep-related problem scores.

After analysis for multivariate effects, unintentional injuries and suicidal thoughts still remained significant for boys, while higher CDI anhedonia scores in girls were associated with parental war involvement (Table 3). Father’s employment and home ownership were not included in the analysis, since these variables depended on veteran status.

**Table 3:** Independent predictors associated with parental war participation: a logistic regression model (forward stepwise)

Predictor variable	B	Standard error	Wald	df	*P*	Exp(B), odds ratio	95% confidence interval
Boys:*							
I often think about killing myself	1.61	0.55	8.65	1	0.003	5.06	1.72-14.89
unintentional injuries:							
never			9.57	2	0.008	1	
1-2 times	-0.68	0.24	8.02	1	0.005	0.51	0.32-0.81
3 times or more	0.15	0.4	0.14	1	0.71	1.16	0.53-2.54
Girls:^†^							
CDI anhedonia^‡^	0.09	0.04	4.7	1	0.030	1.1	1.01-1.19

*χ^2^_3_ for the final model=21.793, *P* < 0.000; Nagelkerke R^2^=0.082.

†χ^2^_1_ for the final model=4.96, *P* =0.026; Nagelkerke R^2^=0.019.

‡CDI – Children’s Depression Inventory.

## Discussion

This study showed an association between parental war involvement and negative psychosocial outcomes, particularly in boys. It did not corroborate the previous findings that parental war participation was associated with lower socioeconomic status, lower employment rate, and homelessness (35-38). Our study showed that children of war veterans were more likely to have unemployed fathers, but were also more likely to come from families with home ownership. This might seem confounding, but can be explained by the Croatian government’s provision of generous benefits to war veterans, especially if they had been wounded, have disabling depression, or suffer from PTSD. Such benefits often include early retirement, financial compensation, and free housing.

Sons of war veterans were significantly more likely to unintentionally injure themselves than sons of non-veterans. According to the literature, unintentional injuries were associated with externalizing disorders such as conduct disorder, oppositional defiant disorder, and attention deficit hyperactivity disorder, as well as internalizing disorders like depression (39-42). Still, this study did not find any connection between parental war participation and depression in male adolescents. Unintentional injuries in this case might be a marker of externalizing behavior, or even some form of parasuicidal behavior as result of an increased propensity towards risk-taking behavior (40,41).

A major finding of this study is the association between parental war involvement and male suicidality across the entire suicidal spectrum (thoughts about death, thoughts about suicide, suicide attempts). Although many adolescents with suicidal ideation and suicide attempts grow up without apparent signs of psychological disturbances, such behaviors are suggested to be indicators and/or predictors of future psychosocial distress. Besides being strong predictors of suicide completion (43,44), suicidal phenomena are indicators of long-term difficulties (45) or concurrent psychiatric disorders like depression (46-48) and conduct/oppositional disorder (49). Also, suicidal ideation has been linked to poorer health outcomes and psychosocial distress (50,51). Longitudinal studies have shown its significant predictive value for a lower level of psychological and social functioning across the lifespan (52-56). Thus, observed suicidal behaviors in early adolescence could be a marker of significant negative outcomes later on in life. This association could be direct and specific, since numerous reports indicate a relationship between suicidality and depression (57-59) and externalizing behaviors or disorder (49,60). We found no evidence that supports such hypothesis, except for unintentional injuries.

Beside possible indications of internalizing and/or externalizing behavior, it is possible that the increased prevalence of suicidal behaviors in sons of veterans might be a consequence of direct identification, imitation, or social learning because suicidality was also prevalent in veterans (61).

We proposed the hypothesis that the association between suicidality and parental war involvement was not so direct and specifically mediated by any distinct psychopathological condition in male adolescents. The presence of suicidality in children of veterans might be linked to the war-induced psychosocial distress of their parents. Active war participation could precipitate a wide range of psychological and psychosocial difficulties in veterans, with indirect effects on their children. Parents, especially fathers who participated in the war, may have incurred psychological distress, which is then transferred onto male children and expressed as suicidality. The mechanism of transfer could be through some form of identification, emotional withdrawal of traumatized fathers, or secondary trauma experienced by the children (9,15). Our finding that male children are more affected by parental war involvement supports the “identification hypothesis,” since the majority of parent veterans were men.

Surprisingly, there was no association between parental war involvement and subjective sleep-related problems. We expected to find this relationship, since suicidality and sleep-related problems might be seen as nonspecific, overlapping indicators of distress. Sleep problems and suicidal ideation are associated with many common psychopathological entities in adolescence (54,62,63), and available reports indicate a link between suicidality and sleep problems in this developmental cohort (64-67).

Our findings should be considered preliminary in the light of several limitations. A major weakness of this investigation is participant assessment and the definition of parental war involvement. Unfortunately, a great proportion (128/803) of participants did not report parental war participation. This could be a source of considerable bias, since it is possible that this subgroup represents those individuals whose families were most severely influenced by the war. However, we do not believe this to be the case. The participation in the war was perceived as an act of defense and patriotism. Therefore, we postulate that children of parents who did not participate in the war were more likely to comprise the group of children who did not report parental war involvement.

As already mentioned, this study used a cross-sectional convenience sample and did not determine causal relationships, which could be assessed only with an appropriately designed prospective follow-up study. The data were based only on adolescent self-reports and were not proofed by using other sources. This could affect the reliability of the results. However, as already mentioned, Lie scores were low, and self report investigations showed that 30-60% of adolescents did not reveal suicidal attempts to anyone, and up to 90% of such cases were unknown to parents (68). Because of this, self-reported data might even be seen as a strength of the study in the sense of better disclosure of internalizing problems. Also, this investigation did not include possible parental psychopathology, so it remains unknown whether mental disorders, particularly depression and PTSD, might have influenced the results. Certainly, there are many more factors that could act as the mediators of transference of war-influenced psychosocial distress, such as the involvement of grandparents or other relatives. However, we believe that this influence is more distal than that from parents.

To conclude, this study found associations between parental war involvement and several observed psychosocial domains of their children. These associations seem to have sex-specific patterns, such as that male adolescents more often reported unintentional injuries and suicidal ideation. At the same time, female adolescents were more resilient and less likely to respond to parental war involvement in the psychosocial domains examined by this study, except for anhedonia which in the absence of raised overall CDI scores is not clinically significant.

Adolescent sons of war veterans constitute a target group for selective prevention. We suggest a development of group and sex-specific, individually tailored preventive interventions for children of war veterans. Possible interventions might focus not only on children but also on war veterans teaching them the importance of parenting skills. This could be done through the National Program of Psychosocial Help for War Veterans (69), which has a broad network of professionals who are mainly helping to resolve veterans’ psychosocial problems and legal status. We propose the inclusion of psychoeducation and consulting about parenting in the routine agenda of these institutions.
